# Structured lifestyle modification and biological aging: effects on PhenoAge, sclerostin, and GDF-15 in arab adults with prediabetes

**DOI:** 10.3389/fragi.2026.1809697

**Published:** 2026-09-10

**Authors:** Ahmed Alenezi, Shaun Sabico, Abdullah M. Alnaami, Syed D. Hussain, Mohamed S. Elrobh, Nasser M. Al-Daghri

**Affiliations:** 1 Chair for Biomarkers of Chronic Diseases, Department of Biochemistry, College of Science, King Saud University, Riyadh, Saudi Arabia; 2 North Medical Tower Hospital, Arar, Saudi Arabia; 3 Department of Biochemistry, College of Science, King Saud University, Riyadh, Saudi Arabia

**Keywords:** biological aging, GDF-15, lifestyle intervention, phenoage, sclerostin

## Abstract

**Background:**

Lifestyle modification programs (LMP) such as diabetes prevention programs may influence phenotypic aging trajectories, but evidence in Middle Eastern populations remains limited. This study examined the effects of a structured LMP on phenotypic age (PhenoAge) as the primary endpoint and circulating sclerostin (SOST) and growth differentiation factor-15 (GDF-15) as secondary endpoints in a cohort of Arab adults with prediabetes.

**Participants and Methods:**

In this randomized clinical trial (RCT), a total of 181 adults (130 males, 51 females; mean age 50.8 ± 10.9 years, body mass index, BMI 30.0 ± 4.8 kg/m^2^) were enrolled. Participants were allocated to either a structured LMP or Control Group (CG) and followed for 6 months. The LMP group was requested to reduce weight by 5%, moderate exercise (150 min/week), reduce fat intake (30%) and increase fiber intake (15g/1 kcal). CG was given brochure. PhenoAge was calculated and assessed alongside anthropometrics, blood biomarkers (HbA1c, fasting glucose, albumin, creatinine, GDF15, SOST), and dietary parameters at baseline and after 6 months.

**Results:**

Compared with the CG, participants in the lifestyle modification program (LMP) showed a significant reduction in PhenoAge (−4.9 vs. +1.3 years; between-group *p* < 0.001). Significant between-group improvements were also observed in weight (−5.7 kg), BMI (−2.0 kg/m^2^), waist circumference (−6.3 cm), and HbA1c (−0.4%; all *p* < 0.001). Neither GDF-15 nor SOST demonstrated a significant intervention-specific effect.

**Conclusion:**

A structured lifestyle intervention significantly improved PhenoAge, accompanied by improvements in metabolic parameters in Arab adults with prediabetes. Changes in GDF-15 and SOST appeared time-dependent rather than intervention-specific. These findings support lifestyle modification as a potential strategy to slow a composite clinical measure of biological aging. ClinicalTrial Registration (NCT06440681).

## 1 Introduction

It is accurate to claim that worldwide, people in modern times are living longer. In fact, according to the World Health Organization (WHO), the global population aged 60 years and above is projected to increase from one billion in 2020 to 1.4 billion in 2030, and to double further to 2.1 billion by 2050 (Ageing and Health, 2026). While the rising prevalence of the geriatric population reflects significant advances in healthcare and longevity, extended lifespan also increases the risk of chronic and debilitating age-related conditions. A recent meta-analysis of 49 studies involving more than 700,000 adults aged >60 years reported a global pooled prevalence of multimorbidity at 46% (Zhu et al., 2025), reinforcing the need for geroscience-driven approaches that target shared aging mechanisms underlying chronic disease accumulation (Kennedy et al., 2014).

Given the heterogeneity of the ageing process, composite clinical biomarker algorithms have emerged as robust indicators of biological aging and predictors of morbidity and mortality (Cohen et al., 2013; Levine et al., 2018). Among these, PhenoAge is a second-generation phenotypic/clinical biomarker algorithm designed to reflect physiological dysregulation and mortality risk (Levine et al., 2018). In brief, PhenoAge was developed using NHANES III training data, where a proportional hazard penalized regression model was applied to reduce 42 candidate biomarkers to a final set of nine biomarkers [albumin, creatinine, glucose, C-reactive protein (CRP), lymphocyte, mean cell volume (MCV), red cell distribution width (RDW), alkaline phosphatase (ALP) and white blood cells (WBC)] in combination with chronological age (Levine et al., 2018). Early clinical trials that used PhenoAge as endpoints such as the CALERIE study indicated promising but non-significant slowing down of aging through long term caloric restriction (Waziry et al., 2023), as well as the DO-HEALTH trial, demonstrating protective effects of omega-3, vitamin D and exercise in age-acceleration (Bischoff-Ferrari et al., 2025). Notably, both trials were conducted in apparently healthy adults, raising the possibility that the effects of such interventions on slowing phenotypic aging could be greater in higher-risk populations, such as those with prediabetes. Despite growing evidence linking healthier lifestyles with reduced biological age acceleration, much of the existing literature remains observational, limiting causal inference and often focusing on generally healthy or Western populations.

Lifestyle factors such as diet, physical activity, and body composition are major determinants of cardiometabolic health and are increasingly recognized as modulators of biological aging (García-García et al., 2024; Song et al., 2025; Sheldon et al., 2026). Indeed, interventional studies suggest that weight loss, improved diet quality, and increased physical activity may reduce biological age acceleration (Qiao et al., 2020), but evidence remains limited, especially in underrepresented regions such as the Middle-East where premature biological ageing is closely linked to cardiometabolic risk factors (Al-Daghri et al., 2022; Al-Attas et al., 2010; Thanaraj et al., 2025). Furthermore, while structured lifestyle modification programs (LMPs) are known to improve metabolic outcomes and are expected to favorably influence aging-related processes, the magnitude of their effect on biological age has yet to be empirically determined in certain high-risk populations.

In addition to phenotypic aging markers, circulating biomarkers such as growth differentiation factor-15 (GDF-15) and sclerostin (SOST) have been associated with aging and metabolic dysfunction. GDF-15 is a stress-responsive cytokine whose circulating concentrations increase with age and are associated with mitochondrial dysfunction, inflammation, cardiometabolic dysfunction, and mortality (Pence, 2022). These associations provided a rationale for investigating whether metabolic improvements induced by lifestyle modification might be accompanied by changes in circulating GDF-15, although intervention studies involving physical activity indicate that its response may be context- and stimulus-dependent (Quist et al., 2023; Kleinert et al., 2018). SOST, predominantly secreted by osteocytes, is an inhibitor of canonical Wnt signaling with an established role in bone remodeling and emerging associations with age, adiposity, diabetes, and bone-muscle-metabolic crosstalk (Guo et al., 2025; Gennari et al., 2012; Alramah et al., 2024). Because both weight loss and physical activity may influence circulating SOST (Tsourdi et al., 2025), it was included as a complementary marker of the skeletal-metabolic axis of aging, while recognizing that the expected direction of change was less clearly established.

Taken together, the measurable effects of LMPs on validated biological aging markers, particularly PhenoAge, and related aging-associated biomarkers such as GDF-15 and SOST, remain insufficiently characterized. Therefore, the present randomized clinical trial aimed to determine whether a structured 6-month LMP can modify biological aging trajectories in Arab adults with prediabetes using PhenoAge as the primary endpoint and GDF-15 and SOST as complementary secondary biomarkers. By integrating phenotypic clocks and emerging biomarkers of ageing, this study also aims to contribute to the emerging body of evidence supporting the use of biological aging markers as modifiable endpoints in lifestyle-based interventions.

## 2 Materials and methods

### 2.1 Study design and participants

**FIGURE 1:**
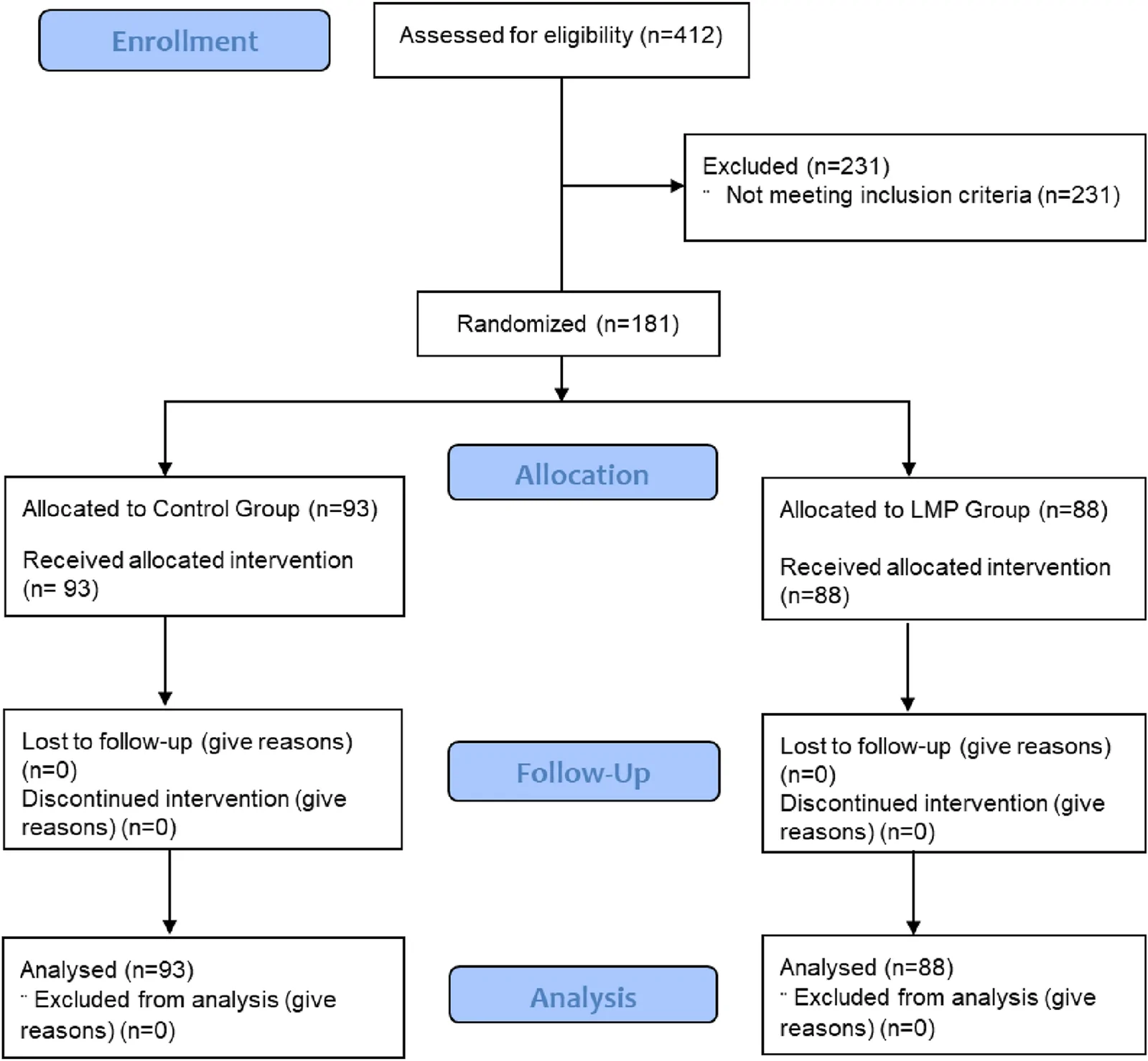
Flowchart of participants.

This multi-center RCT was conducted across primary healthcare centers (PHCCs) in the Arar region, Saudi Arabia. Inclusion criteria comprised adult Saudi men and women aged ≥30 years with confirmed prediabetes defined according to the American Diabetes Association (ADA) criteria (HbA1c 5.7%–6.4%) (American Diabetes Association Professional Practice Committee for Diabetes 2, 2026). Exclusion criteria included a diagnosis of type 1 or type 2 diabetes and any medical condition that could limit full participation in the intervention. Pregnant and lactating women, children and non-Saudis were also excluded. Participants attended their nearest PHCC after an overnight fast for prediabetes screening and baseline assessments. The participant flowchart is shown in Figure 1.

All participants provided written informed consent prior to enrollment. Ethical approval was obtained from the Ministry of Health Northern Borders Health Cluster (NIC-IRB-024–06-11) and King Saud University (E−24–8995). The study was registered at ClinicalTrials.gov (NCT06440681) and was reported in accordance with the Consolidated Standards of Reporting Trials (CONSORT) guidelines for RCTs.

#### 2.1.1 Randomization and study groups

Following screening, eligible and consenting participants were randomly allocated to study groups using a computer-generated sequence produced with the RAND function in MS Excel to either a structured LMP or Control Group (CG)**.** The LMP group received structured educational sessions on diabetes prevention, lifestyle counseling and follow-up, while the CG received standard general advice. While the randomization sequence was computer-generated, no additional allocation concealment mechanism was implemented.

#### 2.1.2 Intervention

The 6-month structured LMP was implemented at participating PHCCs and was adapted from previously validated Saudi lifestyle intervention studies for adults with prediabetes, aimed to prevent diabetes (Al-Hamdan et al., 2021; Wani et al., 2020). In brief, participants assigned in the LMP group were monitored to achieve ≥5% reduction in baseline body weight (kg) by engaging in moderate physical activity (≥150 min/week), limiting total fat intake to ≤30% of total energy and saturated fat to ≤10% and increasing dietary fiber intake to ≥15 g per 1,000 kcal. Participants received individualized dietary counseling from a registered nutritionist every 2 weeks, along with structured 6 education sessions twice a month for 3 months, aimed at increasing knowledge about diabetes and its prevention. Online support was provided throughout the intervention period for the LMP group. The CG received only printed educational materials and were asked to return after 6 months for follow-up tests (Al-Hamdan et al., 2021; Wani et al., 2020).

### 2.2 Data collection

All participants completed a generalized questionnaire containing demographic information and family history. Anthropometric assessments were conducted using standardized procedures at baseline and after 6 months. Measurements included weight (kg) and height (cm) for body mass index (BMI, kg/m^2^) calculation as well as neck circumference (cm). Waist (cm) and hip circumference (cm) for waist-to-hip ratio (WHR) calculation. Systolic and diastolic blood pressure (mmHg) were measured twice at 15 min intervals and the average was recorded.

#### 2.2.1 Sample collection

Fasting venous blood samples were collected at baseline and after 6 months following an 8–10-h overnight fast. Samples were processed and centrifuged, and serum aliquots were stored at −80 °C until analysis. Routine biochemical tests, including screening, were conducted at the Arar Central Hospital laboratory, Arar, Saudi Arabia, a certified and accredited hospital by the Saudi Central Broad for Accreditation of Health Institutions (CBAHI). The remaining fasting serum samples/aliquots were appropriately labeled, stored, and transported to the Chair for Biomarkers of Chronic Diseases (CBCD), Department of Biochemistry, College of Science, King Saud University (KSU), Riyadh, Saudi Arabia, for the analysis of circulating SOST and GDF-15. Transport procedures were executed in accordance with standardized biospecimen handling established by the Ministry of Health in Saudi Arabia.

#### 2.2.2 Sample analysis

Routine biochemical parameters [fasting glucose (mmol/L), glycated hemoglobin (HbA1c %), albumin (g/dL), alkaline phosphatase (ALP) (U/L) and creatinine (mg/dL) were quantified using the Dimension EXL 200 analyzer (Siemens Healthineers, Erlangen, Germany). Complete blood count (CBC) was assessed using the Sysmex XN-1000 device (Sysmex Corp, Hyogo, Japan). C-reactive protein (CRP) (mg/L) was measured using the BN ProSpec NEPH 630 system (Siemens Healthineers, Erlangen, Germany). Serum SOST and GDF-15 concentrations were measured using commercially available assay kits (CUSABIO, Wuhan, China and R&D Systems, Minneapolis, United States of America, respectively), following the manufacturers protocols. The intra- and inter-assay CVs were (2.1% and 10.8% for SOST and 2.27% and 5.44% for GDF-15, respectively). All assays and tests were conducted using standardized automated analyzers with routine internal and external quality control procedures.

#### 2.2.3 Primary endpoint

PhenoAge was calculated and collected at predefined study visits (baseline and 6-month follow-up) using the published algorithm by Levine et al. (Levine et al., 2018), implemented through a publicly available spreadsheet-based calculator (downloaded 26 October 2025) to ensure reproducibility (https://zenodo.org/records/10067628). The phenotypic age algorithm was based on clinical biomarkers (fasting glucose, albumin, creatinine, ALP, CRP, CBC parameters and chronological age in years), as described previously (Levine et al., 2018).

### 2.3 Statistical analysis

Data was analyzed using SPSS version 16.0 (SPSS Inc., Chicago, IL, United States of America). All randomized participants were included in the analysis according to their assigned groups, as no participants were lost to follow-up. Categorical variables were presented as N (%). Normal continuous variables were presented as mean ± SD and non-normal continuous variables were presented as Median (Quartile 1 – Quartile 3). Baseline differences in groups were determined using Chi-square test for categorical variables and independent Student’s t-test and Mann-Whitney U test for normal and non-normal continuous variables respectively. Repeated measures analysis of variance (ANOVA) was used to determine main (group and time) and interaction effects. Non-normal variables were log transformed prior to parametric analyses. Since no baseline differences were elicited, no adjustments were done. A p-value <0.05 was considered significant.

## 3 Results

A total of 181 Saudi adults with prediabetes (130 males, 51 females) out of 412 individuals screened successfully completed the intervention duration. The over-all mean age was 50.8 ± 10.9 years, mean BMI was 30.0 ± 4.8 kg/m^2^, and mean HbA1c was 5.9% ± 0.2. Table 1 shows the baseline characteristics of both groups and showed no differences in age, anthropometrics, marital and education status as well as medical and family history. It is worthy to note that the over-all baseline PhenoAge (years) was slightly younger than the actual age (49.1 ± 12.4). Majority of the participants were married (86%) and had university education (42%). Half of the participants had medical history of hypertension (50%) followed by dyslipidemia (23.3%) and the overwhelming majority (73.1%) had family history of type 2 diabetes. Table 2 shows the baseline biochemical characteristics of both groups and also showed no statistically significant difference across all measured parameters.

**TABLE 1:** Baseline demographic and anthropometric characteristics of participants according to groups.

Parameters	All	CG	LMP	P-values
	181	93	88	
M/F	130/51	69/24	61/27	​
PhenoAge (years)	49.1 ± 12.4	49.8 ± 11.6	48.4 ± 13.2	0.45
Age (years)	50.8 ± 10.9	51.2 ± 10.6	50.3 ± 11.3	0.58
Weight (kg)	81.5 ± 12.0	82.7 ± 12.1	80.2 ± 11.8	0.17
BMI (kg/m^2^)	30.0 ± 4.8	30.5 ± 4.3	29.3 ± 5.1	0.10
Waist-hip ratio	0.95 ± 0.03	0.95 ± 0.03	0.95 ± 0.03	0.89
Neck (cm)	39.7 ± 4.3	39.7 ± 3.0	39.7 ± 5.3	0.97
Systolic BP (mmHg)	124.6 ± 13.9	125.5 ± 11.3	123.6 ± 16.2	0.36
Diastolic BP (mmHg)	80.3 ± 8.3	80.3 ± 8.7	80.2 ± 7.8	0.95
Marital status (%)				
Single	17 (9.4)	12 (12.9)	5 (5.7)	0.13
Married	157 (86.7)	79 (84.9)	78 (88.6)	
Widowed	7 (3.9)	2 (2.2)	5 (5.7)	​
Educational status (%)				
Illiterate	12 (6.6)	4 (4.3)	8 (9.1)	0.27
Read write	17 (9.4)	11 (11.8)	6 (6.8)	
Primary	25 (13.8)	16 (17.2)	9 (10.2)	
Secondary	51 (28.2)	23 (24.7)	28 (31.8)	
University	76 (42.0)	39 (41.9)	37 (42.0)	​
Medical history (%) (n = 90)				
Heart disease	17 (18.9)	7 (14.3)	10 (24.4)	0.66
Hypertension	45 (50)	25 (51.0)	20 (48.8)	
Dyslipidemia	21 (23.3)	13 (26.5)	8 (19.5)	
Thyroid	3 (3.3)	2 (4.1)	1 (2.4)	
GDM	1 (1.1)	0	1 (2.4)	
Others	3 (3.3)	2 (4.1)	1 (2.4)	​
Family history (%) (n = 104)				
Type 1 diabetes	20 (19.2)	13 (23.6)	7 (14.3)	0.48
Type 2 diabetes	76 (73.1)	38 (69.1)	38 (77.6)	
Thyroid disease	8 (7.7)	4 (7.3)	4 (8.2)	

Data presented as Mean ± SD, for normal parameters; N (%) is used to present categorical variables. Significance set at p < 0.05. No covariate adjustment was performed as groups were balanced at baseline on all measured parameters. Abbreviations: SD, standard deviation; BMI, body mass index; GDM, gestational diabetes mellitus.

**TABLE 2:** Baseline biochemical characteristics and intake of participants according groups.

Parameters	All	CG	LMP	P-values
	181	93	88	
HbA1c (%)	5.9 ± 0.2	5.9 ± 0.2	5.9 ± 0.2	0.29
Glucose (mmol/L)	5.9 ± 1.6	6.1 ± 1.6	5.7 ± 1.5	0.11
WBC (10^3^/µL)	7.3 ± 1.9	7.2 ± 1.7	7.4 ± 2.0	0.43
Lymphocytes (%)	38.0 ± 8.5	38.8 ± 9.2	37.1 ± 7.5	0.17
MCV (fL)	84.5 ± 6.1	84.0 ± 6.6	85.0 ± 5.5	0.29
RCD width (%)	13.5 ± 1.6	13.5 ± 1.5	13.4 ± 1.6	0.59
Albumin (g/dL)	3.8 ± 0.5	3.9 ± 0.5	3.8 ± 0.5	0.21
Crea (mg/dL)	0.9 ± 0.2	0.9 ± 0.2	0.9 ± 0.3	0.16
ALP (U/L)	79.5 ± 28.4	78.4 ± 21.1	80.7 ± 34.6	0.60
CRP (mg/L)	2.5 (2.5–6.2)	2.5 (2.5–6.8)	2.5 (2.5–5.7)	0.59
GDF15 (pg/mL)	788.0 (530–1203)	750.0 (578–1134)	789.2 (466–1222)	0.80
SOST (pg/mL)	162.3 (78.5–237.8)	135.7 (77.4–214.1)	185.6 (80.2–252.4)	0.62
Intake (Kcal)	2048.6 (1830–2239)	2110.1 (1908–2289)	1984.1 (1765–2203)	0.14

Data presented as Mean ± SD, for normal parameters; N (%) is used to present categorical variables. Significance set at p < 0.05. No covariate adjustment was performed as groups were balanced at baseline on all measured parameters. Abbreviations: SD, standard deviation; Q1, Quartile 1; Q3, Quartile 3; HbA1c, Hemoglobin A1c; WBC, white blood cell count; MCV, mean corpuscular volume; RCD, width, Red Cell Distribution Width; ALP, alkaline phosphatase; CRP, C-Reactive Protein; GDF15, Growth Differentiation Factor 15; SOST, sclerostin.

### 3.1 Primary and secondary endpoints

For the primary endpoint, there was a significant interaction effect (effect size = 0.217; p < 0.001). Post hoc comparisons showed that LMP group’s PhenoAge significantly improved with a mean 4.9years ±6.3 younger at the end of the program (95% CI: −6.2 to −3.7; p < 0.05 for within group change). CG’s PhenoAge increased by 1.3 ± 5.6 years at the end of the study but this was not statistically different at baseline (95% CI: 0.1 to 2.5; p = 0.035 for within group change). Between group comparisons showed a clinically significant difference in favor of LMP with an estimated decrease of 6.2 years (−4.9 vs. +1.3 years; between-group p < 0.001). For secondary endpoints there was no significant interaction effects for either SOST (effect size = 0.001, p = 0.80) or GDF15 (effect size = 0.002, p = 0.60). Exploratory analysis showed that circulating SOST levels in CG showed a substantial median increase of 100.8 pg/mL (from 135.7 to 236.5 pg/mL), while the LMP group showed a smaller median increase of 12.8 pg/mL (from 185.6 to 198.4 pg/mL). Although the LMP group showed less pronounced SOST elevation than CG, the difference was not statistically significant. For GDF15 levels, the CG demonstrated a median decrease of 159.7 pg/mL (from 750.0 to 590.3 pg/mL), while the LMP group showed a larger median decrease of 224.9 pg/mL (from 789.2 to 564.3 pg/mL). Similar to SOST levels, the difference between groups was not statistically significant. Both groups showed reductions in GDF15, but the intervention did not produce a statistically significant difference (Table 3). Lastly, there was a strong, significant positive correlation between log-transformed GDF15 with PhenoAge at baseline (r = 0.56, p < 0.001). In contrast, log-transformed SOST demonstrated only a modest, inverse correlation (r = −0.18, p = 0.05) (not shown in table). No significant associations between longitudinal changes in PhenoAge and GDF-15 (r = 0.103, p = 0.26) as well as SOST were found (r = −0.357, p = 0.07) (not shown in table).

**TABLE 3:** Anthropometrics and blood parameters according to groups.

Variable	CG		LMP		P-values		
	Baseline	Follow-up	Baseline	Follow-up	Time p	Group p	Time × group p
PhenoAge (years)	49.8 ± 11.6	51.1 ± 12.7	48.4 ± 13.2	43.5 ± 12.1*	<0.001	0.01	<0.001
Weight (kg)	82.7 ± 12.1	84.3 ± 12.8**	80.2 ± 11.8	74.5 ± 9.6**	<0.001	<0.001	<0.001
BMI (kg/m^2^)	30.5 ± 4.3	31.1 ± 4.6*	29.3 ± 5.1	27.3 ± 4.5*	<0.001	<0.001	<0.001
Waist (cm)	100.6 ± 9.5	101.2 ± 9.7	100.3 ± 11.1	94.0 ± 6.8*	<0.001	0.006	<0.001
WHR	0.9 ± 0.0	0.9 ± 0.0	0.9 ± 0.0	0.9 ± 0.1	<0.001	0.39	0.10
Systolic BP (mmHg)	125.5 ± 11.3	127.9 ± 9.1*	123.6 ± 16.2	119.1 ± 9.0	0.201	<0.001	<0.001
Diastolic BP (mmHg)	80.3 ± 8.7	82.8 ± 8.9*	80.2 ± 7.8	76.5 ± 5.7*	0.288	0.002	<0.001
Neck (cm)	39.7 ± 3.0	40.2 ± 2.9*	39.7 ± 5.3	37.7 ± 2.4*	0.003	0.010	<0.001
HbA1c (%)	5.9 ± 0.2	6.0 ± 0.4*	5.9 ± 0.2	5.5 ± 0.2*	<0.001	<0.001	<0.001
Glucose (mmol/L)	6.1 ± 1.6	6.3 ± 1.8	5.7 ± 1.5	5.1 ± 1.3*	0.168	<0.001	0.003
WBC (10^3^/µL)	7.2 ± 1.7	7.1 ± 1.9	7.4 ± 2.0	7.3 ± 2.3	0.58	0.31	0.85
Lymphocytes (%)	38.8 ± 9.2	38.2 ± 8.0	37.1 ± 7.5	36.5 ± 8.5	0.387	0.09	0.98
MCV (fL)	84.0 ± 6.6	86.0 ± 5.6	85.0 ± 5.5	85.7 ± 5.6	0.002	0.64	0.16
RCD width (%)	13.5 ± 1.5	13.3 ± 1.2	13.4 ± 1.6	13.1 ± 1.4	0.008	0.41	0.82
Albumin (g/dL)	3.9 ± 0.5	4.2 ± 1.0*	3.8 ± 0.5	4.9 ± 1.6*	<0.001	0.005	<0.001
Creatinine (mg/dL)	0.9 ± 0.2	1.1 ± 0.7	0.9 ± 0.3	0.9 ± 0.3	0.04	0.06	0.24
ALP (U/L)	78.4 ± 21.1	88.1 ± 26.5	80.7 ± 34.6	84.8 ± 20.6	<0.001	0.87	0.17
CRP (mg/L)	2.5 (2.5–6.8)	7.4 (4.8–13.8)	2.5 (2.5–5.7)	7.5 (1.7–13.5)	0.03	0.37	0.83
GDF15 (pg/mL)	750.0 (578–1134)	590.3 (448.3–760.7)	789.2 (465.7–1221.6)	564.3 (453.3–733.9)	<0.001	0.76	0.60
SOST (pg/mL)	135.7 (77.4–214.1)	236.5 (80.3–380.4)	185.6 (80.2–252.4)	198.4 (118.7–389.6)	<0.001	0.73	0.80
Dietary intake (Kcal)	2110.1 (1908–2289)	2160.8 (2012–2294)	1984.1 (1765–2203)	1794.4 (1612–1951)*	0.03	<0.001	0.003

Data presented as Mean ± SD, for normal parameters and median (Quartile 1 – Quartile 3) for non-normal parameters. * and ** Indicates within-group significance at p < 0.05 and at p < 0.01 respectively. No covariate adjustment was performed as groups were balanced at baseline on all measured parameters. Abbreviations: SD, standard deviation; Q1, Quartile 1; Q3, Quartile 3; HbA1c, Hemoglobin A1c; WBC, white blood cell count; MCV, mean corpuscular volume; RCD, width, Red Cell Distribution Width; ALP, alkaline phosphatase; CRP, C-Reactive Protein; GDF15, Growth Differentiation Factor 15; SOST, sclerostin.

**FIGURE 2:**
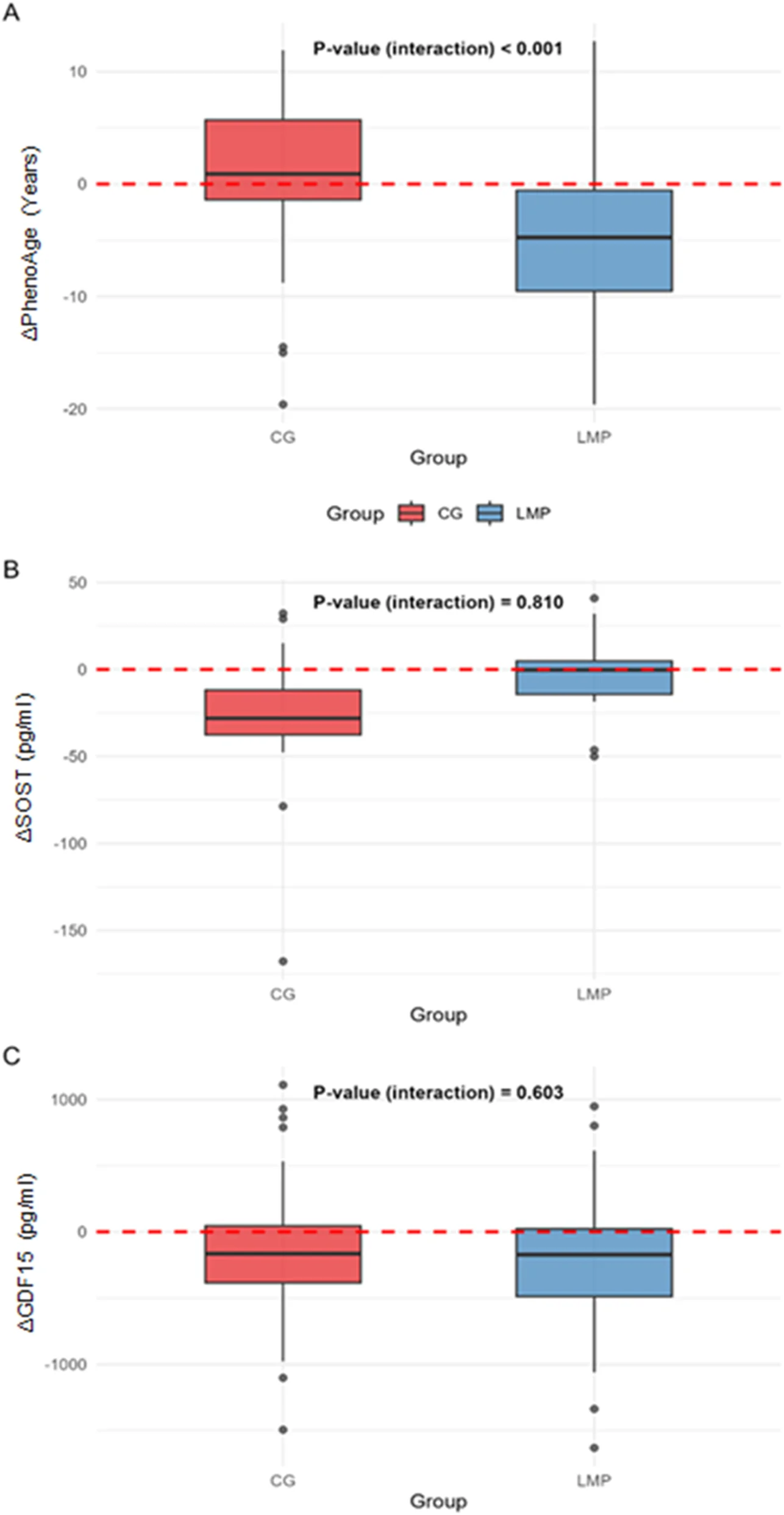
Changes in **(A)** PhenoAge **(B)** SOST **(C)** GDF15 among CG and LMP groups. Median changes were calculated as Follow-up - Baseline.

Figure 2 shows the plotted between-group differences in individual change scores obtained for (Δ) for PhenoAge (2 A) (p < 0.001), SOST (2 B) (p = 0.81), and GDF-15 (2C) (p = 0.60) across the intervention period (p-values obtained from Table 3).

### 3.2 Effects on anthropometric and clinical parameters

**FIGURE 3:**
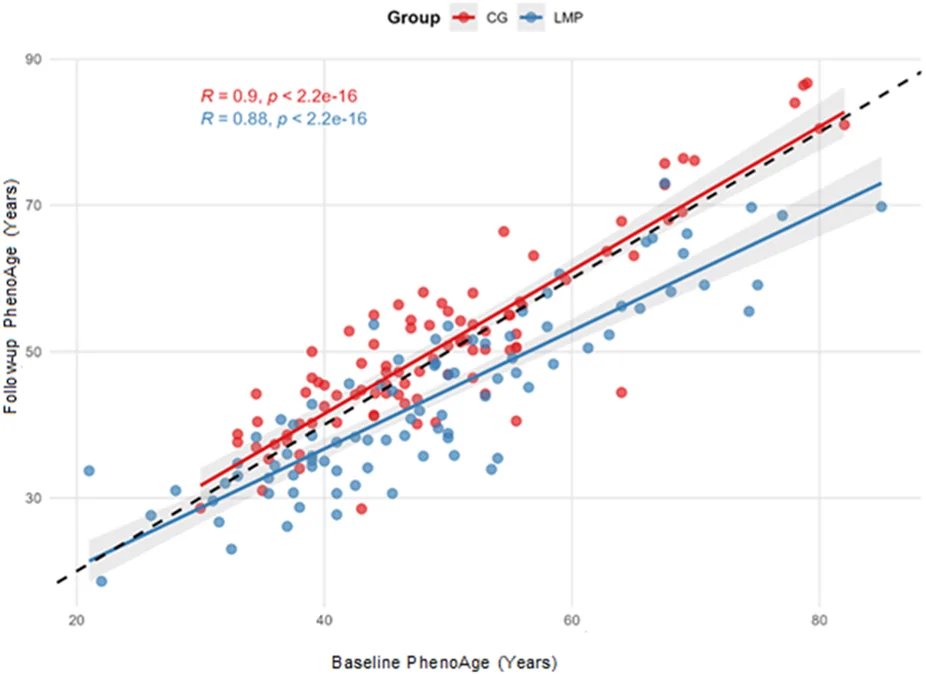
Scatterplot between PhenoAge at baseline and follow-up among CG (red dash) and LMP (blue dash) groups.

Within-group comparisons showed a significant increase in most anthropometric measures in the CG, including weight, BMI, systolic and diastolic BP as well as neck circumference. In parallel, significant improvements were seen overtime in the LMP group, most notably in weight, BMI, waist, neck circumference and diastolic BP (p-values <0.05). Between group comparisons showed a clinically significant difference in weight, BMI, waist, neck circumference and blood pressure in favor of the LMP group (p-values <0.001). In terms of glycemic parameters, between group comparisons also showed a clinically significant difference in the LMP group in both HbA1c (p < 0.001) and fasting glucose (p = 0.003). Among the parameters included in the calculation of PhenoAge, only albumin showed a clinically significant difference, again in favor of the LMP group (p < 0.001) with the effect size of 0.076. The rest of the comparisons are shown in Table 3. Lastly, Figure 3 shows the equally strong significant correlations between the PhenoAge at baseline and follow-up of both CG and LMP groups.

### 3.3 Adherence indicators

The LMP group showed substantially higher achievement of the 30% fat reduction goal (21.6% vs. 9.7%, p = 0.044) and both goals combined (8.0% vs. 1.1%, p = 0.059) compared to CG, while fiber goal achievement was similar between groups (35.2% vs. 32.3%, 0.791). The LMP group achieved a significantly higher rate of 5% weight reduction (78.4%) compared to CG (4.3%) (p < 0.001). Furthermore, the LMP group achieved a significant increase in walking minutes (median 60 vs. 0 at baseline), while CG remained unchanged (interaction p < 0.001). However, the LMP group still fell short of the 150-min weekly goal.

## 4 Discussion

The present RCT demonstrated that a 6-month structured LMP can significantly reduce biological aging, as measured using a composite biological aging marker (PhenoAge), and these changes were parallel to the established improvements in cardiometabolic factors such as weight, glycemic control, and blood pressure. To the best of our knowledge, this is the first RCT in a homogeneous Arab population with prediabetes to evaluate a validated phenotypic aging marker as the primary endpoint and other emerging biomarkers of aging (GDF-15 and SOST) in response to a structured LMP. Both GDF-15 and SOST showed temporal changes with no significant intervention-specific effects, suggesting distinct biomarker-specific responses to the intervention. Unlike prior observational studies reporting associations between lifestyle behaviors and biological aging, the present study provides interventional evidence from a RCT demonstrating that a structured LMP can significantly modify a validated biological aging marker in a high-risk Arab prediabetes cohort.

Previous intervention studies have shown that weight loss, caloric restriction, and increased physical activity can reduce age acceleration (Kou et al., 2025; Van Damme et al., 2026). Trials such as the CALERIE study and other lifestyle interventions have reported modest reductions in aging markers following dietary or behavioral interventions (Waziry et al., 2023). Similarly, observational studies have linked higher physical activity and healthier diets to improve metabolic age (Izquierdo et al., 2025; Nagata et al., 2024). Our findings extend this evidence to a Middle Eastern cohort, supporting the universality of improved lifestyle effects on slowing biological aging, especially on populations at higher risk for chronic conditions such as those with obesity and prediabetes.

The significant clinical reduction in PhenoAge in the LMP group likely reflects improvements in systemic metabolic regulation, inflammation, and physiological resilience. PhenoAge is a clinically derived composite measure of phenotypic aging that integrates chronological age with multiple physiological biomarkers (Levine et al., 2018). Unlike DNA methylation-based aging clocks (Hannum et al., 2013; Horvath, 2013; He et al., 2020), the PhenoAge algorithm used in the present study does not require direct measurement of DNA methylation. Therefore, the observed change represents a composite clinical measure of phenotypic aging rather than direct evidence of altered epigenetic aging. It is worthy to note that both PhenoAge and leukocyte telomere length (LTL), an established biomarker of cellular aging, are both observed to be favorably responsive to lifestyle and dietary interventions (Carvalho et al., 2025), and are modestly associated with one another as they capture overlapping yet distinct biological processes of ageing (Pearce et al., 2022). Regardless, given that PhenoAge reflects metabolic and inflammatory pathways associated with cellular senescence, partly driven by LTL attrition, the inclusion of both validated and established aging biomarkers in RCTs may provide more robust evidence to facilitate their translation into clinical practice.

As for the secondary endpoints, GDF-15 has consistently been associated with cardiometabolic risk factors, aging, chronic disease, and mortality (Teramoto et al., 2024; di Candia et al., 2021; Abubaker et al., 2025). Reductions in GDF-15 are typically observed following improvements in metabolic status or reductions in systemic stress (Eggers et al., 2008). In the present RCT, both groups showed modest but non-significant temporal changes, and GDF-15 decreased over time in both groups without a significant time × group interaction, indicating that the observed changes cannot be specifically attributed to the lifestyle intervention.

Similar to GDF-15, SOST has also been associated with aging, adiposity, and metabolic disorders (Guo et al., 2025; Gennari et al., 2012; Alramah et al., 2024; Stelmaszek et al., 2025). While most of the research on SOST focus on its role in skeletal health, new evidence suggests that crosstalk between bones and muscles (including cardiac muscles), are indirectly influenced by osteocrine factors such as SOST (Li et al., 2025). In the present study, the observed increase over time without between-group differences may reflect underlying bone or metabolic remodeling independent of the intervention. Furthermore, as a novel biomarker, SOST still has no widely accepted clinical cut-offs, with values varying according to assay used and bone status, the latter of which was not assessed in the study. Cumulatively, the observed divergence of findings between PhenoAge estimates and secondary endpoint biomarkers, GDF-15 and SOST, highlights the complexity and multifactorial nature of aging pathways. Whereas PhenoAge reflects integrated, multisystem aging processes, GDF-15 and SOST represent more specific biological pathways and may exhibit distinct, stimulus-dependent responses. Intervention studies suggest that circulating GDF-15 responses to exercise are heterogeneous and context-dependent, with acute increases and variable longer-term responses depending on the population and intervention characteristics (Quist et al., 2023; Kleinert et al., 2018; Yıldırım Ayaz et al., 2025). Similarly, SOST responses may depend on competing effects of weight loss and reduced skeletal loading versus exercise-induced mechanical stimulation (Armamento-Villareal et al., 2012). Thus, the absence of significant intervention-specific effects for GDF-15 and SOST, despite their baseline associations with PhenoAge, may reflect differences in biomarker responsiveness and supports their complementary roles in phenotypic aging assessment.

The authors acknowledge several limitations. Findings of the present RCT may have limited ethnic and geographic generalizability beyond similar populations. The assessment of GDF-15 and SOST at only two time points limited our ability to characterize their temporal response patterns and distinguish intervention-related responses from physiological variability. As the study focused solely on its endpoints, potential factors that may confound results such as detailed dietary composition and types of physical activity were not included. Despite the caveats, the study, to our knowledge, is the first RCT in the Arab region to use a validated composite biological aging measure as a primary endpoint to assess the effects of real-world lifestyle interventions used in the region. The randomized controlled design, together with the integration of phenotypic aging and emerging age-related biomarkers, further adds to the novelty of the study. Finally, incorporating telomere length alongside biological aging markers such as PhenoAge could provide complementary insight, and this will be considered in future studies.

## 5 Conclusion

A 6-month structured LMP was associated with a significant reduction in PhenoAge among Arab adults with prediabetes, suggesting that short-term improvements in cardiometabolic health may be reflected in a composite clinical measure of phenotypic aging. SOST and GDF-15 exhibited time-dependent but not intervention-specific changes. These findings support the potential role of lifestyle interventions in improving multi-system health and modifying clinical indicators associated with aging in populations at increased cardiometabolic risk. Future trials incorporating multiple longitudinal assessments may help clarify the responsiveness and temporal trajectories of GDF-15 and SOST to lifestyle interventions.

## Data Availability

The original contributions presented in the study are included in the article/supplementary material, further inquiries can be directed to the corresponding author.
